# Supplementary material for: Identification and functional analysis of non-coding regulatory small RNA FenSr3 in Bacillus amyloliquefaciens LPB-18
Source: PeerJ. 2023 May 15;11:e15236. doi: 10.7717/peerj.15236 (PMC10194069; doi:10.7717/peerj.15236)
Supplement: Supplemental Information 4 [file peerj-11-15236-s004.zip › KO/CK-vs-T1_map/map04072.html]

KEGG PATHWAY: Phospholipase D signaling pathway - Reference pathway


|  |  |
| --- | --- |
| **Phospholipase D signaling pathway - Reference pathway** |  |

[
Pathway menu
| Organism menu
| Pathway entry
| Show description
| User data mapping
]

Phospholipase D (PLD) is an essential enzyme responsible for the production of the lipid second messenger phosphatidic acid (PA), which is involved in fundamental cellular processes, including membrane trafficking, actin cytoskeleton remodeling, cell proliferation and cell survival. PLD activity can be stimulated by a large number of cell surface receptors and is elaborately regulated by intracellular factors, including protein kinase C isoforms, small GTPases of the ARF, Rho and Ras families and the phosphoinositide, phosphatidylinositol 4,5-bisphosphate (PIP2). The PLD-produced PA activates signaling proteins and acts as a node within the membrane to which signaling proteins translocate. Several signaling proteins, including Raf-1 and mTOR, directly bind PA to mediate translocation or activation, respectively.

|  |  |  |
| --- | --- | --- |
| Reference pathway | 184% 150% 122% 100% 82% 67% 55% | 图片下载 |
